# iHAT: interactive Hierarchical Aggregation Table for Genetic Association Data

**DOI:** 10.1186/1471-2105-13-S8-S2

**Published:** 2012-05-18

**Authors:** Julian Heinrich, Corinna Vehlow, Florian Battke, Günter Jäger, Daniel Weiskopf, Kay Nieselt

**Affiliations:** 1 VISUS, University of Stuttgart, Allmandring 19, 70569 Stuttgart, Germany; 2 Integrative Transcriptomics, ZBIT, University of Tübingen, Sand 14, 72076 Tübingen, Germany

## Abstract

In the search for single-nucleotide polymorphisms which influence the observable phenotype, genome wide association studies have become an important technique for the identification of associations between genotype and phenotype of a diverse set of sequence-based data. We present a methodology for the visual assessment of single-nucleotide polymorphisms using interactive hierarchical aggregation techniques combined with methods known from traditional sequence browsers and cluster heatmaps. Our tool, the interactive Hierarchical Aggregation Table (iHAT), facilitates the visualization of multiple sequence alignments, associated metadata, and hierarchical clusterings. Different color maps and aggregation strategies as well as filtering options support the user in finding correlations between sequences and metadata. Similar to other visualizations such as parallel coordinates or heatmaps, iHAT relies on the human pattern-recognition ability for spotting patterns that might indicate correlation or anticorrelation. We demonstrate iHAT using artificial and real-world datasets for DNA and protein association studies as well as expression Quantitative Trait Locus data.

## Background

Genome wide association studies (GWAS) are used to study the variation of genes between individuals (the genotype), and their association with a variety of complex traits (the phenotype), e.g. diabetes, heart disease, or arthritis. GWAS have become an established method to alleviate the identification of genetic risk factors of diseases, as they make use of recent technologies that allow a rapid and cost-effective analysis of genetic differences. Within the last five years, many single-nucleotide polymorphisms (SNPs) could be identified with the help of GWAS, implicating hundreds of loci for common traits [1]. The huge amount of data produced by GWAS poses a great challenge for data analysis and visualization. Here, we use interactive hierarchical aggregation in heatmaps together with a sequence alignment view as a tool for the visual analysis of correlations between sequence data and associated metadata.

Heatmaps [2] and other table-based visualizations display values of a data table using color to show patterns in the data [3]. This approach is flexible and can be used with all kinds of data, such as expression data [2,3], sequence data [4,5], or graphs [6]. Heatmaps are often combined with dendrograms for both rows and columns (usually obtained from hierarchical clustering) that serve two purposes: (1) they explicitly visualize the hierarchy of rows or columns and (2) implicitly determine their ordering. While table-based visualizations are useful to obtain an overview of a dataset and to visually find patterns that might be difficult to spot with automatic methods, there are two drawbacks of sequence alignment viewers or heatmaps: First, the patterns that emerge depend on the order of rows and columns. For time-series expression data and sequence alignments, column order is usually fixed and hence does not pose any problem. Where applicable, row and/or column order can be partly defined (i.e. within groups) using hierarchical clustering. Second, the number of data items that can be visualized is restricted by the number of pixels that are available. As a consequence, many datasets produced today cannot be visualized in a single image using the traditional tools. Many implementations provide a scrolling mechanism that enables the user to adapt the region of interest, but this approach does not provide an overview of the data. Hierarchical aggregation [7] can be used to reduce the number of data items that have to be rendered and navigation with fixed hierarchies has been implemented for heatmaps [8], graphs [6], financial data [9], and databases [10]. In the context of biological data visualization, some sequence viewers provide grouping of rows [11,12] and columns [13,14]. However, user-driven aggregation and interactive construction of hierarchies for biological data has, to our knowledge, not been investigated yet. While heatmaps use colored matrices to illustrate data values of a table, sequence viewers use them to show aligned sequences of nucleic acids or amino acids. Color is employed to indicate the type of nucleic acid or amino acid, or it represents some attribute of the alignment. There are many sequence viewers with different extents of functionality [5]. While tools like JBrowse [15] and the human genome browser [16] mainly serve as sequence viewing software, the Integrative Genomics Viewer [17] further supports import and visualization of microarray data and genomic annotations. CINEMA [11], ClustalW/ClustalX [18], Jalview [14], and STRAP [12] visualize (multiple) sequence alignments and the latter three allow phylogenetic tree computation either during or after the alignment. SeaView [19] further enables the user to construct and investigate phylogenetic trees of alignments using different algorithms for tree construction. Slack *et al*. [13] use an information visualization technique called "accordion drawing". The aim of their hyperbolic scaling (focus+context) approach is to guarantee visibility of user-defined (or otherwise selected) columns in a zoomed-out view of a large sequence alignment by reducing the level of detail of other parts of the alignment.

Here, we present the interactive hierarchical aggregation table (iHAT) to combine the visualization of sequences (genotype) and expression data (phenotype) to support genetic association studies. Similar to traditional heatmaps, iHAT consists of a table-based visualization of both primary sequence data and metadata with an attached dendrogram. In contrast to the tools mentioned in the previous section, color maps and aggregation strategies in iHAT both depend on the type of data and aggregation state for every column individually, such that columns of type nominal, ordinal, ratio, and interval use different aggregation functions and are mapped to different (single- or multi-hue) color maps. Rows and columns can be aggregated using either the attached hierarchy or by interactive selection. To find associations between genotype and phenotype, the aggregation process can be guided by metadata representing the phenotypes.

We demonstrate our techniques with multiple sequence alignments of DNA and amino acid sequences with attached phenotype metadata. For the analysis of DNA data, we use the IEEE VAST 2010 Challenge data [20]. For proteins, we use amino acid sequence data of the neuraminidase protein of 15 H5N1 influenza virus samples [21]. To illustrate the column aggregation of metadata we analyze and visualize expression Quantitative Trait Locus (eQTL) data. This paper extends our previous work on iHAT [22] and uses original material thereof.

## Methods

This section presents the general framework for interactive hierarchical aggregation and the design choices we made for iHAT.

### Terminology

We consider multivariate data as a set of *N samples *(rows), each comprising the same number of *values *(columns) from a set of *M variables*. Each variable has a *scale type *[23], which can be one of:

*• Nominal*: Nominal data has neither ordering nor metric. Only the equality operation (=) is defined for values on this scale.

*• Ordinal*: Ordinal data defines an ordering. In addition to the operations allowed for nominal data, the order operator (<) is defined.

*• Interval*: Interval data is measurable on an interval scale. In addition to the operations defined for ordinal data, the difference operator (-) is defined.

*• Ratio*: For data on a ratio scale, the equality of ratios can be established. In addition to the operations defined for interval data, the division operator (/) is defined.

**Figure 1:**
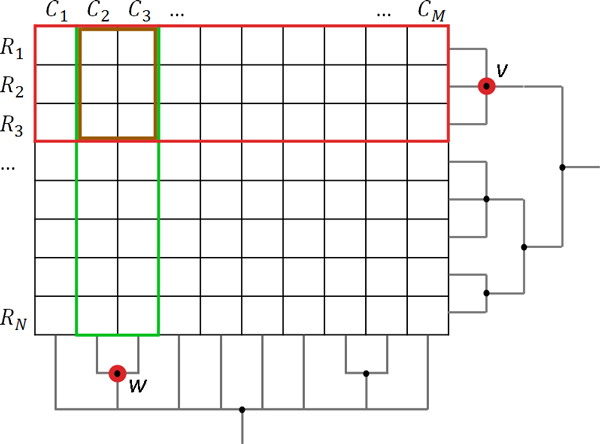
**Aggregation scheme**. Multivariate data is represented as a table of *N *rows and *M *columns, where each column can be of a different scale type. Rows and columns can be aggregated independently, resulting in hierarchies as indicated by the attached dendrograms. Aggregation of rows is applied column-wise, according to the respective scale type, while the aggregation of columns is only supported for compatible scale types. For example, the set of leaf nodes of *v *is a collection of rows *R_v _*and the set of leaf nodes of *w *is a collection of columns. Aggregating *v *results in a new row with values determined using rows *R*_1_, *R*_2_, and *R*_3 _(red square). Similarly, aggregating *w *results in a new column *C_w _*with values determined from columns *C*_2 _and *C*_3 _(green square). As a result of collapsing both *v *and *w*, the overlap of the red and green squares will be represented by only one cell.

Column *C_j _*contains all values of variable *j *and row *R_i _*contains all values of the sample *i *(see Figure 1). The value of a cell at row *i *and column *j *can now be addressed using either *R_i, j _*or *C_j,i_*. Rows and columns can independently be aggregated into a tree *T *= (*V*, *E*) with vertices *V *and edges *E*, resulting in hierarchical multivariate data. Using the terminology from Elmqvist and Fekete [7], our multivariate samples are *data items *that can be grouped into *aggregate items*. While both data and aggregate items are represented by a vertex *v *∈ *V*, data items define the set *L*={*v*∈*V*|*succ*(*v*)=∅} of leaf nodes and aggregate items define the complement *I = V\L *of interior nodes plus the root node. Aggregate items can also be grouped, such that the root node denotes the set of all data items.

Starting with a "flat" table, row and column hierarchies are initialized with a tree of height one, where every row/column is a leaf-node and child of the root. Aggregation produces new internal nodes for which a value (i.e. a list of values) has to be computed. These values are the results of an *aggregation function *that can be different for every internal node. The aggregation function depends on several factors, including the scale type of the aggregated items, their distribution, or the degrees of freedom for the visual representation of an aggregate (area, color, texture, etc.). Choosing the appropriate aggregation function therefore is an important part of the design choices to be made for the problem at hand and will be discussed for iHAT in the next section.

Another aspect to consider is the choice of color maps applied to the values of all nodes in the tree. One might use different mappings for leaf-nodes and for internal (aggregated) nodes to distinguish between both types and to provide a visual hint of where the user is currently navigating within the hierarchy. Depending on the underlying data type, internal nodes might carry information about the local properties of the tree, such as the number of immediate children, the number of leaf-nodes, the height in the tree, etc., which can also be visualized using color.

### Design choices

As described in the previous section, aggregation of rows and columns into hierarchies requires several design choices to be made, as there are many different approaches to realize the general principle. In this section, we therefore provide the design choices made for our implementation iHAT.

The motivation for iHAT was to join sequence views with heatmaps to provide a visualization for association studies. To communicate this separation, we decided to split the general table layout into two aligned views: the sequence view containing biological sequences with a fixed alphabet represented as nominal data and a separate heatmap view for the mostly ratio-scaled metadata, usually representing the corresponding phenotypes.

#### Color coding

iHAT maps values to color depending on their scale type. Since the appropriate color map greatly depends on the data that is visualized [24], we adopted general design principles from the visualization literature [25,26] for the different scale types. In the heatmap view, ratio-scaled values are colored using a single-hue color map with varying saturation. For nominal columns, we adapt the number of different hues to the number of classes contained in the respective column and map the relative frequency of the *consensus *(the most frequent child item) to saturation. In this way, the color scheme is used to visualize the (un-)certainty of the consensus.

**Figure 2:**
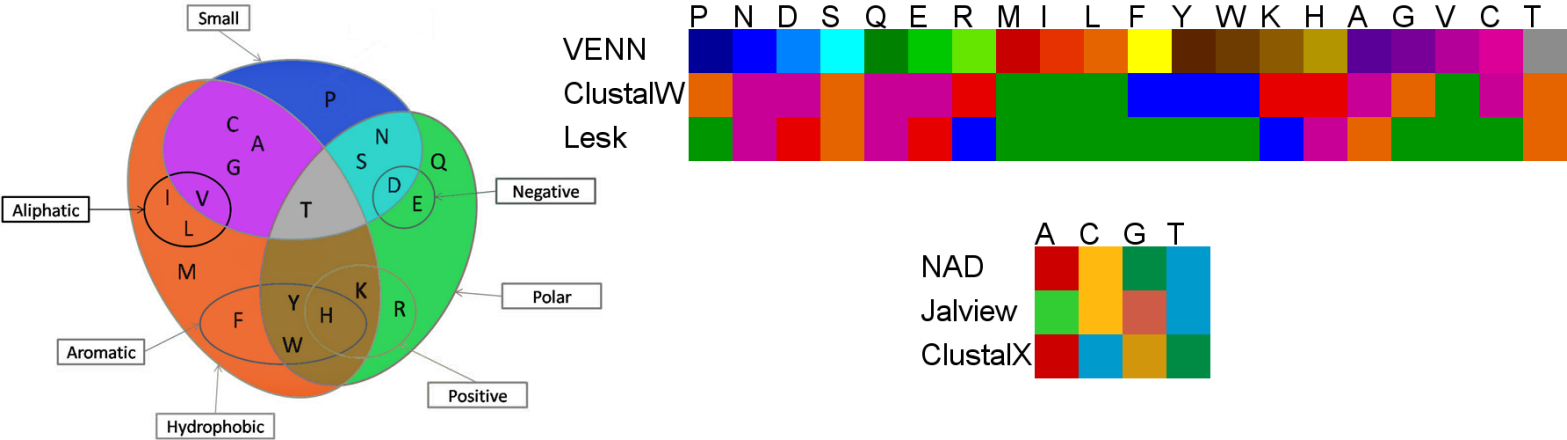
**Color scheme**. Left: Venn diagram grouping of amino acids based on the biochemical properties: hydrophobicity, size and polarity. Colors for the intersection groups are derived by additive blending of the colors of respective properties. Right, top: Alternative color schemes for amino acids (note that the scheme labeled *ClustalW *is based on the default coloring without color parameter file with N, D, Q, E, A and C mapped to purple.) Right, bottom: Alternative color schemes for nucleic acids.

Nucleic and amino acid sequences are interpreted as nominal variables for which iHAT offers color maps used by tools like ClustalX [18], Jalview [14], Lesk [27], or the Nucleic Acid Database [28]. In addition, we developed a novel color map for amino acids following the Venn diagram [29] grouping of amino acids, only considering the groups formed by the three main properties: hydrophobicity, size, and polarity (see Figure 2). Based on these properties and their intersections, the Venn diagram divides amino acids into seven groups. Amino acids are thus colored with respect to the group to which they belong, where each group is assigned a color. All amino acids within the same group are mapped to slight variations of the respective color of this group (see Figure 2), with maximum difference within the groups. This newly developed color scheme helps the user with getting an immediate impression of the biochemical properties of amino acids within the sequences.

#### Hierarchical aggregation

**Figure 3:**
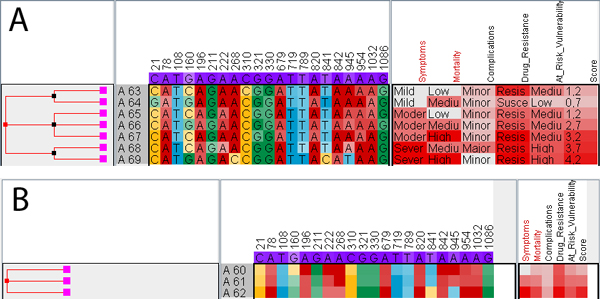
**Row hierarchies**. Internal nodes of the hierarchy can be collapsed resulting in consensus rows (which are assigned unique numerical labels starting with a capital 'A'). For nominal values, the relative frequency of the character in the consensus is mapped to saturation of the respective color. For ratio-scaled values, the mean is used instead. The row-hierarchy has been created using the automatic aggregation feature: (A) internal nodes at depth one correspond to rows with the same *symptoms *with children grouped by *mortality*. (B) Collapsing internal nodes at level 2 show the consensus of rows with the same value for *symptoms*. Hiding labels improves the visual pattern matching due to uncluttering, as we can discover columns with the same trend of saturation.

In iHAT, a table is used to render the visual representations of multivariate samples while the data hierarchy is visualized with a dendrogram attached to the rows of the table (see Figure 3). Although aggregation of columns is possible in general, we decided to allow hierarchies only for metadata columns, as there was no practical implication for column aggregation in the sequence view. Furthermore, we do not render dendrograms for columns in order to better use the given screen real estate. For multivariate data without existing hierarchy, we create a tree of height one, where every sample is a child node of the root and a leaf node of the tree.

iHAT implements bottom-up aggregation: a hierarchy can be constructed by aggregating a set of selected samples (rows that represent leaves in the aggregation tree) or aggregates (rows that represent internal nodes). Several consensus rows (internal nodes) can also be joined into a new consensus row. Interactively constructed trees can be exported (in Newick format) and imported again for further investigation. The dendrogram itself is visualized as (left-to-right) node link diagram. To reduce the number of rows and to compare subclasses of the hierarchy, internal nodes can be collapsed to show a consensus row or expanded to show all underlying samples of the aggregate individually.

Given the tabular layout of visual items and visual aggregates, we use color to convey information about the distribution of items. Depending on the color space, color can be split into further variables such as hue, saturation, and value or red, green, and blue, which gives more degrees of freedom for the design of visual aggregates. However, as a simple mapping of aggregate variables to these color changes very likely interferes with the coloring principles outlined in the previous section, we use the following data-type dependent strategies to assign aggregate values to colors.

For nominal data, we use multi-hue bivariate color maps to indicate class membership and map saturation (with constant value) to the relative frequency of the consensus. We use the HSV color space [30] to choose the final color: The hues required to distinguish classes can be chosen by distributing all classes over the range of available hues. This strategy enables one to use saturation as an indication for the uncertainty of the most frequent child item. While this approach can easily be automated, it does not scale well for a large number of classes. For instance, the color scheme used for amino acids (as introduced in the previous section) allows the user to differentiate between groups of amino acids, whereas differences within a group are less prominent.

Ordinal data is treated similarly to nominal data with respect to aggregation strategies and color mapping because color maps for ordered data highly depend on the semantics of the data. We use a discrete color table for the ordinal value and represent uncertainty equivalently to nominal values.

Following the design principles for ratio and interval data [26], we are interested in conveying quantitative information using the color channel. Data on a ratio scale is aggregated by computing the mean value of all children. Different color maps exist that ensure that the equivalence of distances of ratios and intervals is perceived correctly. We map ratio values to a univariate single-hue color map, where the ratio value determines saturation.

For interval data, we found that it was most useful to convert it to a ratio scale, as this allows for the computation of the mean value and for using the same color mappings as for ratio-scaled data. Considering that the color of the initial data can be distributed equally over a range of saturations on a single-hue colormap, in-between values (as computed by the mean) are easier to identify by the viewer as for ratio-scaled data.

### Implementation

iHAT was implemented in the Java™programming language. The software and additional information are available at http://www.vis.uni-stuttgart.de/iHAT.

In addition to the design choices presented in the previous sections, iHAT supports sorting and filtering of rows and columns as well as automatic aggregation of rows. As the case studies in this paper make use of these features, a short description thereof is given in the following sections.

#### Sorting and filtering

Rows can be sorted with respect to selected metadata columns. If several metadata items are used for sorting, this results in a nested sorting, which is a useful feature to interactively construct a hierarchy of samples.

**Figure 4:**
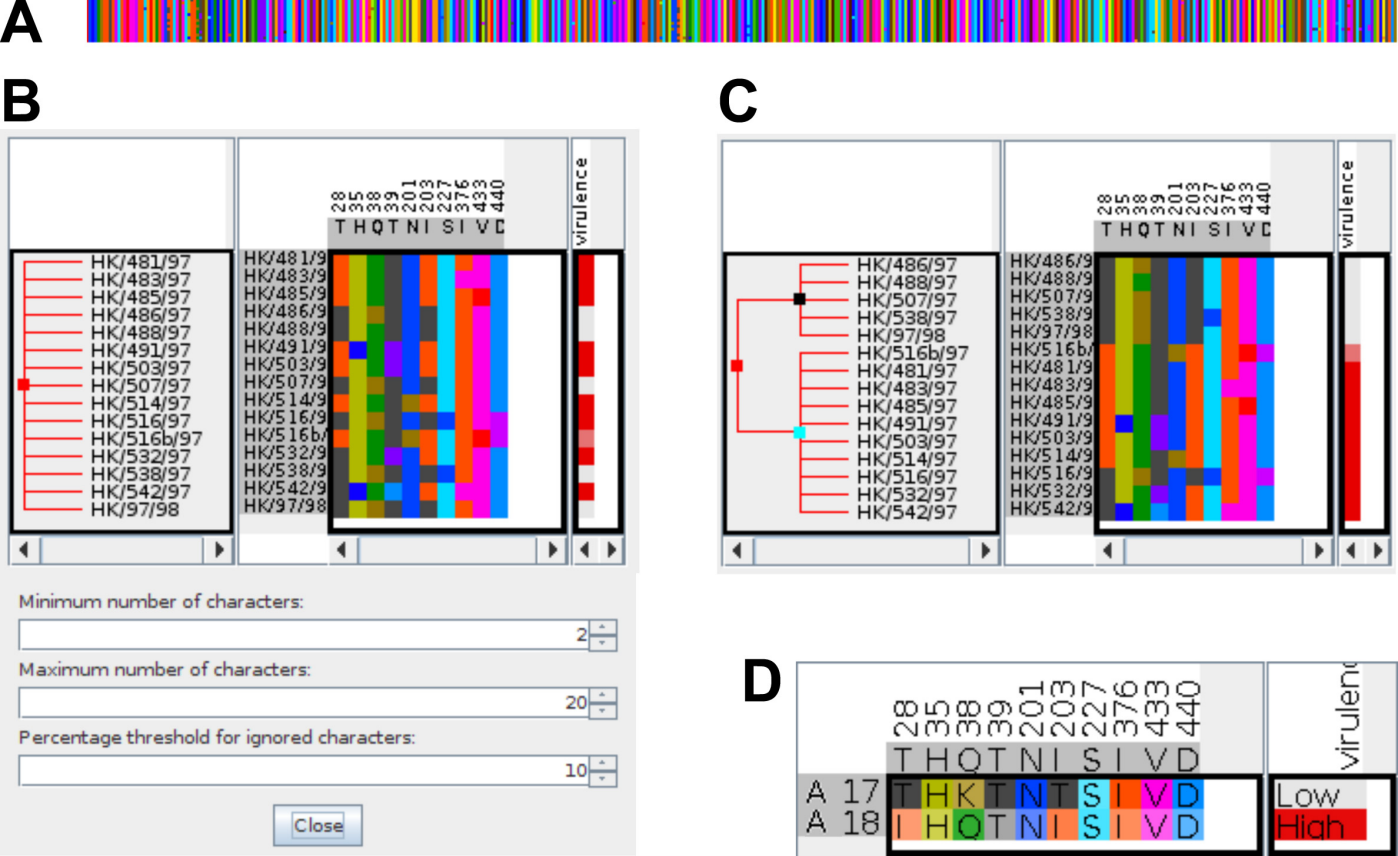
**Interactive hierarchical aggregation for amino acid sequences**. Using iHAT to find sequence positions correlated with virulence in 15 sequences of the neuraminidase protein of H5N1 *influenza *virus samples. (A) the unfiltered alignment using the color map based on Venn diagram grouping; (B) after removing uninformative columns (parameters shown in the dialog window below the alignment), only ten positions remain. (C) sequences were sorted by virulence and internal nodes were added aggregating by 2 levels of *virulence*; (D) after aggregation on (collapsing) the internal tree nodes, the final alignment of the two aggregated sequences (labeled with unique numerical identifiers starting with 'A') clearly shows positions correlated with virulence.

Columns can be filtered to hide uninteresting information. Reasonable filtering options should always be based on the underlying data. Since our application targets sequences of nucleic acids or amino acids (as samples), current filtering options were designed to hide columns that are too homogeneous or too noisy. iHAT further supports semi-automatic filtering of columns in the sequence view, based on the nominal scale-type and the following characteristics:

*• Number of symbols: *The number of different symbols (nucleic or amino acids) is determined considering symbols that exceed a given count in the respective column. Only columns where the number of symbols lies within a specified interval of interest are shown (Figure 4B, first and second option). This supports the process of revealing associations between genotype and phenotype.

*• Missing symbols: *Columns with fewer than the given percentage of unknown symbols (i.e. gaps in the sequence) are shown (Figure 4B, bottom option). Columns that contain mostly gaps (resulting from the alignment) do not contain any information that helps the user find correlations with the phenotype (metadata) and can therefore be hidden. While unique insertions or deletions may convey a difference in phenotype, they should at least occur in a certain percentage of the underlying population to allow statistically meaningful conclusions.

*• Noise: *When searching for associations between genotypes and phenotypes, we are interested in finding columns that show differences between the phenotypes, while being mostly uniform within each phenotype. By using a row-order dependent noise filter, we aim at hiding columns that violate this assumption, i.e. columns that do not match the sorting based on metadata: We count all row indices *i *(1 ≤ *i *<*N*) where the symbol *R_i,j _*differs from the symbol *R_i_*_+1,j _and hide all columns where the percentage of such indices is above a given threshold.

*• Prior knowledge: *Users can supply a list of columns of interest (determined by an external method, e.g. some correlation or other statistical method) and only show those columns.

#### Automatic aggregation

Using iHAT, we found that a common task is to sort rows by one or more metadata columns and aggregate rows with common metadata values. With *automatic aggregation*, iHAT uses selected metadata columns to automatically build the aggregation tree. This is achieved by successively aggregating rows with the same metadata value for all selected columns, in the order of selection. See Figure 3 for an example of automatic aggregation.

## Results

To demonstrate the functionality and usefulness of iHAT, we used it for the analysis of nucleic acid sequences and amino acid sequences with associated metadata. Here, rows represent sequences, columns represent alignment positions, and cells contain nucleic acids (amino acids), or metadata of scale type ratio, interval, nominal, or ordinal. In the matrix view, each position is colored either by nucleic acid (or amino acid) or attribute value. Depending on the scale type, different color schemes are used.

One of the main features of iHAT is the aggregation of rows (here sequences). As sequences are of nominal type, the nucleic acid (amino acid) of the aggregated (consensus) sequence at position *i *is chosen as the one with largest frequency (i.e. the mode), giving rise to the color value in the respective cell. The frequency of the nucleic acid or amino acid in the consensus (i.e. the degree of conservation in the alignment) is mapped to saturation. For ratio values (within metadata), the mean value is taken as the consensus.

When using filtering of columns and sorting and aggregation of rows based on some metadata in combination with colormapping, column specific patterns emerge that facilitate the detailed analysis of correlation between nucleic acid (amino acid) sequences and metadata (e.g. phenotype data). To unclutter the matrix view and to improve the visual pattern matching, labels (for nucleic acids, amino acids or attribute values) can be hidden on demand (see Figure 3).

### Example 1: DNA association

For the analysis of nucleic acid data, our general approach is to associate genotype (sequence) with phenotype data (metadata) with the help of the matrix-based alignment view. We used the IEEE VAST 2010 Challenge data [20] (mini challenge 3: genetic sequences) to demonstrate this approach. The dataset consists of 58 sequences with 1403 nucleic acids each. For every sequence, a set of five attributes describing the associated phenotype is given. Four of these are of scale type ordinal (*symptoms*, *mortality*, *complications*, *at risk vulnerability*) and one of type nominal (*drug resistance*). Since the following analysis is based on a derived value of these attributes, we decided to convert all attributes to ratio scale (Table 1) before loading the data into iHAT.

**Table 1:** Mappings from ordinal and nominal data to the ratio scale.

		ordinal values of the attributes			ratio
symptoms	mortality	complications	drug resistance	at risk vulnerability	scale
mild	low	minor	susceptible	low	0.0
moderate	medium	-	intermediate	medium	0.5
severe	high	major	resistant	high	1.0

**Figure 5:**
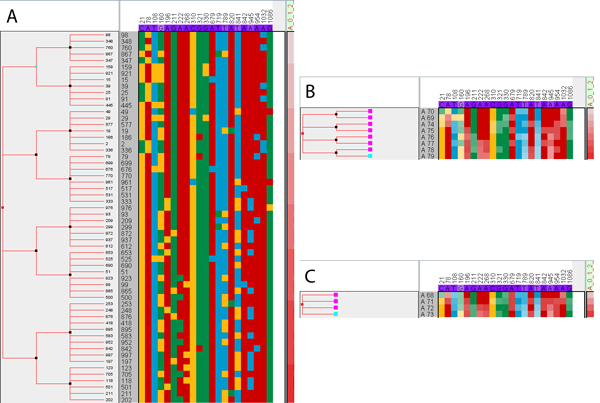
**Aggregation and correlation**. Aggregation of rows and columns can be used to find correlations between sequence data and metadata. Aggregating metadata columns in (A) shows the distribution of "overall virulence", for which the causing SNPs in the sequence are of interest. Aggregating rows (B,C) according to the dendrogram (which was computed in advance for the purpose of demonstration) leads to a condensed view where it is possible to find similar patterns between positions in the sequence and the metadata. For example, column 841 shows an inverse pattern to the "overall virulence". Going back to the expanded view now allows the user to investigate the cause of the patterns, where sequence position 841 shows a binary distribution with many yellow spots in the bottom half and some outliers in the top.

For a detailed explanation of the metadata types and their values, we refer to [20]. Based on the ratio values, we computed a score by aggregating over all metadata columns. The resulting column is computed as the average of all phenotypes, which is a good representation of the "overall virulence" in this application. (Note that in our previous work [22], the same score was computed with an external tool before loading the data into iHAT.) Sorting and aggregating rows based on the aggregated columns visually reveals correlations between phenotypes and specific positions within the sequence (see Figure 5). The sorted table shows "overall virulence" in the rightmost column, indicated by the increasing saturation of red with increasing values. However, it is difficult to find columns in the sequence where this pattern is reflected. The automatic aggregation feature of iHAT allows us to aggregate rows by a user-defined metadata column. Using this feature results in a condensed view where the high variation in different colors is replaced with a high variation of saturation in the individual columns. Here, column 841 seems to express an inverse pattern to the "overall virulence", with decreasing saturation from top to bottom. After an additional row-aggregation step, averaging two levels of "overall virulence", more columns with the same or the inverse pattern can be seen. Column 108 shows the same pattern, while columns 196, 789, 841, and 945 show the inverse pattern. With this information, we can go back and look at the fully expanded table again. Here, we see that column 108 has an increasing number of cytosine (yellow) from top to bottom, but that most of it occurs at low levels of "overall virulence". Column 789, in contrast, appears to have an equal distribution of cytosine at the bottom-half of the table, indicating that this mutation occurs with the same frequency for either low or high virulence and that there is nothing in between. Reversing the column aggregation reveals that the binary attribute "drug resistance" causes this effect (see [22]).

This example demonstrates two important principles: (1) Aggregation can be useful to reduce the visual clutter and with that the cognitive load needed to find patterns. (2) Aggregation is not lossless; moving back to a detailed view is important to investigate the cause of the patterns that were found in the aggregated overview.

### Example 2: Amino acid sequences

To show the application of iHAT to protein sequences, we used sequence data for the neuraminidase protein of 15 H5N1 influenza virus samples [21]. The sequences were aligned using ClustalW [31] and were loaded into iHAT together with the respective strains' virulence strengths (classified as *low*, *intermediate*, or *high*). The complete alignment comprises 450 columns (Figure 4A). We first apply a filter to show only those columns that contain at least two different amino acids, each present in at least 10% of the samples, which drastically reduces the number of columns to inspect (Figure 4B). Then we sorted the sequences according to the virulence annotation and created new internal nodes in the aggregation tree by aggregating all strains with *low *virulence into one group and aggregating the remaining *intermediate *and *high *virulence strains into another group (Figure 4C). Collapsing the aggregation nodes results in our final alignment of two *consensus *sequences. From this alignment, we can clearly see that column 28 (T vs I), 38 (K vs Q), and 203 (T vs I) are correlated with the strength of virulence (Figure 4D). In the original publication, the correlation of column 28 with lethality in mice was experimentally validated.

### Example 3: eQTL

The discovery of genetic variations that are correlated with specific phenotypic outcomes has become of great interest in scientific research. Genome wide-association studies help to identify genome sequence variations that are significantly correlated to specific phenotypes. eQTL studies go one step further. In such studies, additionally to the sequence-based data reflecting the genotypic variations, gene expression values of tens of thousands of genes are measured. The goal is to identify genetic variations that can be significantly associated with differences in gene expression in order to connect certain genotypes with specific phenotypic outcomes.

To illustrate how iHAT can be used to visually analyze eQTL data we applied it to the data set provided for the BioVis 2011 contest [32]. This data set consists of genomic variations for 7555 genomic loci, gene expression data for 15 different genes, and meta-information regarding the disease state ("affected"/"unaffected") for a hypothetical spiked-in disease. Sequence data are available for a total of 500 patients of which 193 are affected. Furthermore, results from a statistical analysis with the whole genome data analysis toolset PLINK [33] are also published. For a comprehensive analysis, we loaded the data into iHAT. The resulting primary matrix consisted of 7555 columns, one for each SNP, and 500 rows, one for each patient, respectively. Each cell in the matrix encodes for one of the three possible states:

1. both alleles are identical to the reference

2. one of the two alleles differs from the reference

3. both alleles differ from the reference

These three states are encoded in iHAT using the colors green, yellow, and red, respectively. In addition to the SNP data, we also loaded gene expression data as metadata into iHAT together with the patients' affection states. The metadata matrix consists of 15 columns that represent the expression values of the 15 genes and one column for the affection state. The color encoding for the affection state is chosen such that cells are colored red if the patient is affected and white otherwise. For the gene expression data, we chose a blue-white-red color gradient to encode for down-regulation, no regulation, and up-regulation, respectively.

**Figure 6:**
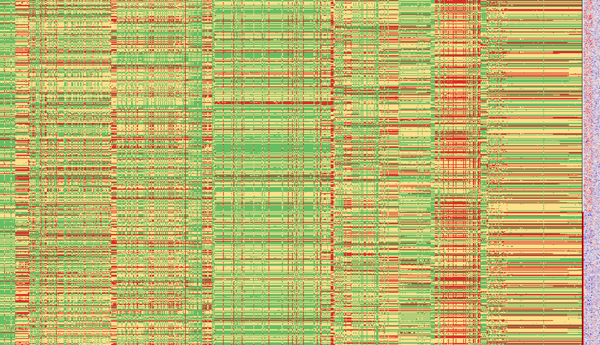
**Visualization of eQTL data**. iHAT visualization of all 696 significant SNPs of all 500 patients together with metadata. Color is used to encode the type of SNP in the respective patient: green = the two alleles are identical to the reference sequence, yellow = one allele differs from the reference, red = both alleles are different with respect to the reference. Next to the SNP matrix, metadata is visualized using color to represent the respective value. The leftmost column shows the phenotype data 'affection', followed by 15 columns of expression data represented by a color-gradient blue-white-red of the 15 genes.

**Figure 7:**
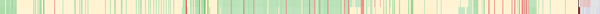
**Row-aggregated eQTL data**. All 696 significant SNPs are shown. Patients were aggregated into two groups according to their affection states.

**Figure 8:**
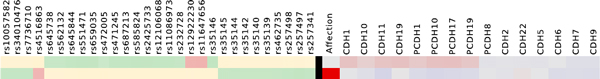
**Aggregated view after visual selection**. Aggregated view showing the 29 remaining SNPs after visual selection. Only SNPs that have a different color in the affected (red) and unaffected (white) patient's group have been selected.

**Figure 9:**
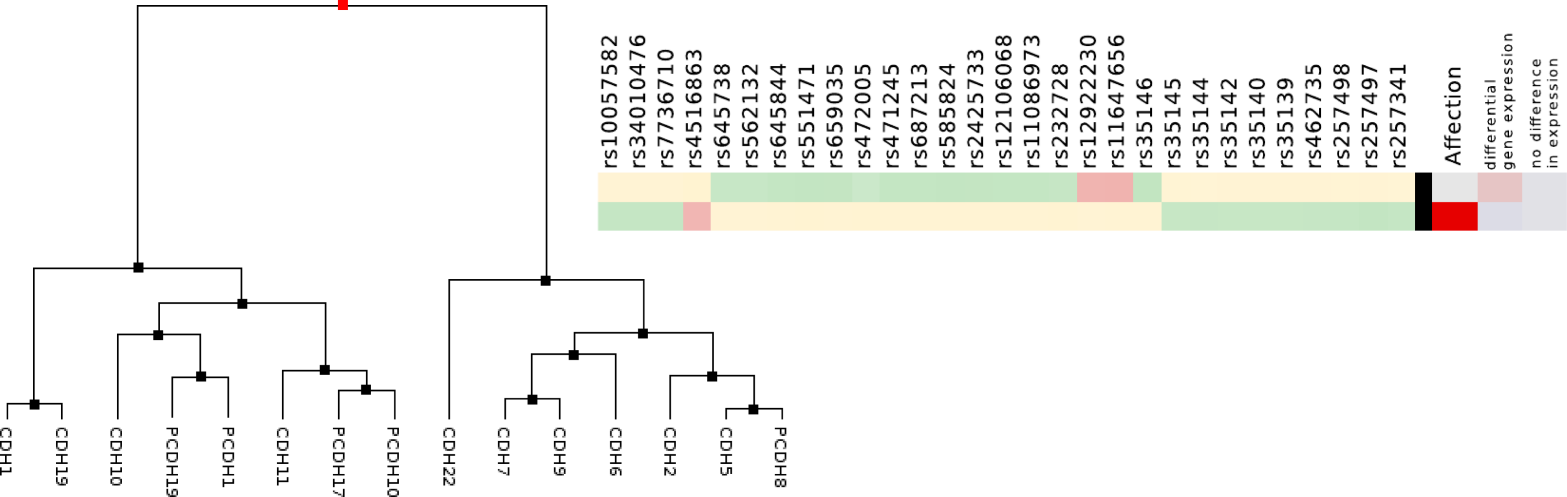
**Hierarchical clustering**. Left: Hierarchical clustering of the 15 genes from the BioVis 2011 contest dataset [32] using the UPGMA method and the Euclidean distance as distance measure (left). Genes are partitioned into two large clusters, namely differentially expressed genes and genes showing no differential expression between affected and unaffected patients. Right: Aggregated visualization in iHAT showing 29 significant SNPs associated with the patients disease states. Patients have been aggregated into the two groups affected (red) and unaffected (white), genes have been aggregated according to the clustering.

To visually analyze only SNPs that significantly influence the gene expression of one of the 15 genes, we applied some pre-filtering steps. First, we removed all SNPs that have an *R*^2 ^value smaller than 0.1 or a *p*-value larger than 0.05 according to the single-locus PLINK results. This resulted in a total of 845 remaining SNPs. We then applied a second filtering based on the two-locus PLINK results. The two-locus results encompass SNP pairs that significantly influence the expression of one of the 15 genes. We used the set of 845 SNPs and filtered for those that are contained in such SNP pairs. This left 696 SNPs in the data set that were used for further visual analysis in iHAT (Figure 6). Since one is interested in significant genomic differences between the affected and unaffected group, we aggregated all affected and unaffected patients, respectively. The result is shown in Figure 7. As can be seen, there are still a lot of SNPs left that do not show different colors for the two groups, although they have a low p-value. We therefore manually selected all those SNPs that show a different color between the affected and unaffected state. A total of 29 different SNPs could be identified in this way. These 29 SNPs are shown in Figure 8. We also conducted a standard test of independence using Pearson's *χ*^2 ^test. For 375 of the 696 SNPs, the null hypothesis was rejected (*p *< 0.05), of which only 13 among the 29 SNPs show a different color between both states. Thus the aggregation step helped identify more putatively relevant SNPs than a mere statistical analysis. These are the SNPs where the majority of patients have different allele combinations between the affected and unaffected groups. In the case of the other SNPs detected only by the statistical test, the majority of the patients in both groups have the same allele combinations. Next, we looked for those genes whose expression is correlated with the disease state. Therefore, we performed a hierarchical clustering of the genes after aggregation of the patients into the two affection groups. The hierarchical clustering was performed using the UPGMA method with Euclidean distance as distance measure. The 15 genes were clearly separated into two distinct groups, which can be seen in Figure 9. The genes were then resorted in iHAT according to the hierarchical clustering. Afterwards, the two groups of genes were aggregated separately resulting in two metadata columns representing the mean expression of the two gene groups for the affected and unaffected patients. After aggregation, the differences in expression between these two gene groups stand out very clearly (Figure 9). Further analyses of the 29 identified SNPs showed that these SNPs are only contained in SNP pairs that in combination are associated with genes differentially expressed between affected and unaffected patients.

Using the Biovis 2011 contest data set we demonstrated iHAT's potential in visually analyzing eQTL data. Due to iHAT's aggregation features, we were able to identify a total of 29 SNPs that are highly associated with the patients disease states. While manual inspection of all 696 SNPs is clearly infeasible (as can be seen in Figure 6) and statistical testing did not reveal all relevant SNPs, the aggregation by affected and unaffected patients showed significantly associated SNPs for visual selection.

## Discussion

There are several issues that have to be discussed. Most importantly, the success of a visual assessment of association studies with iHAT greatly depends on the size and complexity of the dataset at hand. While the VAST Challenge (Example 1) was solved using iHAT exclusively, additional computational methods needed to be incorporated for the other datasets. Due to the complexity of real-world biological data, this is true for most visualizations used in this context. According to the visual-analytics paradigm [34], both interactive visual and automatic methods need to be integrated to achieve the best results. In this course, it is important to note that the hierarchical aggregation framework presented in this work can be readily extended with data-mining techniques, machine-learning algorithms or computational statistics to add new aggregation functions, color maps, preprocessing steps, etc. However, the scalability of the system regarding the ability of human investigators to visually recognize patterns in increasingly large datasets has to be studied further.

## Conclusion

The huge amount of data produced by GWAS implies a great challenge for data analysis and visualization. In particular, scalability and pattern matching problems need to be addressed. Hence, we developed iHAT, which is based on a framework for generic data. iHAT serves the visual analysis of correlations between samples and associated metadata using interactive hierarchical aggregation in combination with a sequence browser.

Our usage scenarios showed that it is particularly useful for the exploration of genomic data, especially if phenotype information is available. iHAT allows the user to aggregate rows and columns interactively, where metadata (phenotype information) can be used to guide this process. The aggregation guided by metadata turned out to be helpful in revealing patterns from a multiple sequence alignment that might have their origin in SNPs related to the phenotype(s) under consideration. Furthermore, the tool can be used to find correlations between mutations within amino acid sequences and some traits (phenotypes). The assessment of phenotypes is further facilitated by column aggregation, as the number of columns to be compared with patterns in the sequence can be reduced.

With iHAT, we present a tool that transforms the problem of correlating genotype with phenotype to a visual pattern matching task. Starting from an overview of the aligned sequences, followed by filtering of uninformative sites and subsequent computation of consensus sequences for chosen subgroups, patterns emerge.

## List of abbreviations used

DNA: Deoxyribonucleic acid; eQTL: expression Quantitative Trait Locus; GWAS: genome wide association study; HSV: Hue Saturation Value; iHAT: interactive hierarchical aggregation table; SNP: single-nucleotide polymorphism.

## Competing interests

The authors declare that they have no competing interests.

## Author contributions

JH developed the basic framework of hierarchical aggregation for data tables used in this work. JH, DW, FB, and KN extended the concept and made the design choices for biological sequences. JH, FB, and KN extended and applied these methods to the IEEE Vast Challenge Data, FB, GJ, and KN analyzed the neuraminidase dataset and CV, FB, GJ, JH, and KN analyzed the IEEE Biovis Contest dataset. CV developed iHAT in the Java™programming language. All authors wrote, read, and approved the final manuscript.
